# Special Issue: “Recent Advances in Hypertension, Hypercholesterolemia and Type 2 Diabetes”

**DOI:** 10.3390/biomedicines14010098

**Published:** 2026-01-03

**Authors:** Tomislav Bulum

**Affiliations:** 1 Department of Diabetes and Endocrinology, Vuk Vrhovac University Clinic for Diabetes, Endocrinology and Metabolic Diseases, Merkur University Hospital, 10000 Zagreb, Croatia; tomobulum@gmail.com; Tel.: +385-12353887; Fax: +385-12331515; 2 School of Medicine, University of Zagreb, Šalata 3, 10000 Zagreb, Croatia

Type 2 diabetes, the most prevalent form of diabetes associated with obesity, remains one of the leading global causes of mortality and disability [1]. In parallel with the increased prevalence of overweight and obesity, there is an increased prevalence of type 2 diabetes. Additionally, due to falling mortality, aging, and population growth, there is a rapidly growing number of global patients with type 1 diabetes, an autoimmune disease usually diagnosed in childhood, especially in lower-income settings [2]. In general, almost 600 million people worldwide are affected by diabetes, with projections of additional rapid growth in the coming decades [3]. It is also noteworthy that the economic burden of diabetes and its chronic complications caused at least USD 1 trillion in health expenditure [3,4].

Type 2 diabetes is usually clustered with other risk factors associated with metabolic syndrome, such as hypertension and hypercholesterolemia. The central pathophysiological condition connecting these diseases is insulin resistance associated with visceral obesity [5]. Due to reduced peripheral insulin sensitivity, β-cells must increase insulin secretion, which can result in β-cell exhaustion with hyperglycemia and development of type 2 diabetes over time. Some genetic and environmental factors can affect islet function and connect obesity and type 2 diabetes [6]. The most serious consequences of diabetes stem from its chronic complications; nowadays, it is the leading cause of blindness among the adult working-age population, chronic kidney disease, and non-traumatic lower limb amputations [7,8,9,10,11].

While microvascular complications of diabetes are an important cause of disability worldwide, myocardial infarction and stroke, which are macrovascular complications, are the predominant causes of premature morbidity and mortality in individuals with diabetes [12]. This elevated cardiovascular risk is largely attributed to the clustering of metabolic risk factors commonly associated with obesity and hyperglycemia, such as hypertension and dyslipidemia [13]. Insulin resistance alters systemic lipid metabolism and leads to the development of a characteristic form of dyslipidemia with high levels of plasma triglycerides, low levels of high-density lipoprotein (HDL), and predominance of small dense low-density lipoprotein (LDL) [14]. Small dense LDL is more atherogenic compared to normal-sized LDL, leading to accelerated atherosclerotic plaque formation. Small dense LDL have increased sensitivity to oxidation, have increased vascular permeability (as small molecules easily penetrate through the endothelium), and undergo conformational changes in Apo B (leading to decreased affinity for LDL receptors), with longer retention of LDL in circulation (making it more liable to oxidative modification and uptake into the blood vessel) [15].

Hypertension is one of the most prevalent chronic conditions worldwide, affecting more than one billion adults and representing the leading risk factor for cardiovascular disease. It markedly increases the risk of heart attack, stroke, heart failure, and kidney disease by placing continuous strain on the heart and blood vessels [16]. Effective blood pressure control is therefore essential for reducing the risk of these serious complications. However, many individuals with hypertension remain undiagnosed, untreated, or inadequately controlled. Strict management of blood pressure, along with optimization of serum lipid levels, body weight, and lifestyle factors such as smoking cessation, is crucial to control the global burden of cardiovascular disease and reduce the risk of premature mortality [17].

Although recent trends indicate a decline in all-cause and cardiovascular mortality, the risk remains high, particularly among patients with diabetes [7]. Thus, further efforts are essential to reduce the burden of comorbidities and lower the risk of premature mortality associated with hypertension, hyperglycemia, and hypercholesterolemia. In this context, this Special Issue brings together the latest research and advancements contributing to the understanding and management of hypertension, hypercholesterolemia, and type 2 diabetes, highlighting novel therapeutic approaches and emerging clinical insights. It comprises eight original research articles that offer a comprehensive perspective on current developments in the field.

The first research article in this Special Issue confirms that both systolic and diastolic blood pressure are independent risk factors for diabetic retinopathy in patients with type 2 diabetes [18,19]. The study included a total of 160 patients with type 2 diabetes, of whom 46.3% had diagnosed diabetic retinopathy. Binary logistic regression analysis revealed that hypertension, longer diabetes duration, and hyperglycemia were the main predictors of diabetic retinopathy, while the effects of systolic and diastolic blood pressure on diabetic retinopathy development remained significant even after adjustment for diabetes duration and glycated hemoglobin levels. No significant differences in lipid profiles or renal function were observed between groups according to diabetic retinopathy severity. The findings of this study underscore the importance of regular monitoring and optimal control of blood pressure in patients with type 2 diabetes to prevent the onset and progression of diabetic retinopathy, which remains the leading cause of blindness among working-age adults.

The second research article aimed to compare pulse wave velocity, a non-dipper profile, and arterial hypertension in subjects with prediabetes and healthy individuals. The gold standard method for assessing arterial stiffness is pulse wave velocity, which is also a proven predictor of target organ damage, cardiovascular disease, and overall mortality [20]. Additionally, hyperglycemia increases pulse wave velocity [21]. This study included a total of 301 subjects, of whom 150 had prediabetes. The highest pulse wave velocity values were observed in patients with uncontrolled hypertension, in those with prediabetes, and in prediabetic non-dippers. Significantly higher pulse wave velocity values were found in subjects with prediabetes and non-dipping profiles regardless of the presence or regulation of hypertension [22]. These results confirm that individuals in the prediabetes stage have a higher cardiovascular risk and a greater likelihood of target organ damage.

The third research article evaluated the impact of changes in body mass index on the incidence of glomerular hematuria in Korean adults. The prevalence of glomerular hematuria in the general population is reported to be as high as 38%, and it serves as a marker of glomerular dysfunction or damage [23]. Obesity is a well-established risk factor for kidney disease through a cluster of metabolic syndrome-related disturbances, such as diabetes, hypertension, and dyslipidemia [24]. In this study, the incidence of recurrent and persistent hematuria associated with glomerular disease was observed in both significant weight loss and weight gain groups even after adjusting for potential confounders. Weight loss greater than 10%, rather than baseline body mass index, was associated with the incidence of recurrent and persistent hematuria in glomerular disease [25]. These findings suggest that maintaining a stable body weight may help prevent recurrent and persistent hematuria related to glomerular disease in Korean adults.

The fourth research article explored single nucleotide polymorphisms of the RAC1 gene as novel susceptibility markers for neuropathy and microvascular complications in type 2 diabetes. Single nucleotide polymorphisms in the RAC1 gene have recently been linked to hyperglycemia and type 2 diabetes [26]. The RAC1 gene is proposed to have at least a causal role in diabetic microvascular complications. This study included 1470 DNA samples from patients with type 2 diabetes and aimed to determine whether the common single nucleotide polymorphisms of the RAC1 gene are associated with diabetic complications such as neuropathy, retinopathy, nephropathy, angiopathy of the lower extremities, and diabetic foot syndrome. The results demonstrated, for the first time, that three RAC1 haplotypes were associated with an increased risk of diabetic retinopathy, neuropathy, and angiopathy of the lower extremities in patients with type 2 diabetes [27]. The RAC1 gene’s polymorphisms might represent novel and sex-specific markers of microvascular complications in patients with type 2 diabetes.

The fifth research article investigated predictors and outcomes of sodium–glucose cotransporter 2 inhibitors (SGLT2i) discontinuation in a real-world population after hospitalization for heart failure. SGLT2i are now fundamental therapeutic options in patients with heart failure and can reduce the risk of hospitalization for heart failure or cardiovascular death [28]. However, treatment with SGLT2i has been discontinued in some patients, leaving them without the favorable benefits of SGLT2i therapy. This study included a total of 159 patients who were hospitalized for heart failure and administered SGLT2i. A lower serum albumin level and a higher dose of furosemide at baseline were independently associated with the future discontinuation of SGLT2i. However, patients who stopped SGLT2i therapy (12% of studied patients) had a 32% incidence of heart failure recurrence or cardiovascular death during the 1-year therapeutic period compared to an 11% risk in those who continued with SGLT2i therapy [29]. These results highlight the importance of initiating and managing SGLT2i therapy in patients with heart failure, particularly in those with lower serum albumin levels and higher doses of furosemide.

The sixth research article examined the types and frequency of mutations in the mitochondrial DNA (mtDNA) nucleotide sequence in hypervariable regions 1 and 2 (HV1 and HV2) and the 12S RNA coding sequence of the D-loop in postmenopausal women with hypertension. Genetic alterations in mitochondrial DNA are associated with a variety of human disorders, including hypertension, diabetes, coronary heart disease, cancer, and endometriosis [30,31]. This study included 100 women, 53 of whom were postmenopausal and 47 of whom were premenopausal. The frequency of nucleotide sequence alterations in mtDNA was substantially higher in postmenopausal women than in premenopausal women. The mtDNA polymorphisms of the nucleotide sequence in the HV1 and HV2 regions, the HV2 region alone, and the 12S RNA coding sequence were associated with estrogen deficiency and a more severe course of hypertension [32]. This new knowledge may facilitate clinical studies investigating the role of hormone replacement therapy as a protective treatment against mtDNA mutation.

The seventh research article investigated the involvement of endogenous enkephalins in glucose homeostasis. Enkephalin and enkephalin analogs increase the intake of palatable and high-fat foods [33]. Enkephalins are found in several peripheral organs, including the pancreas, and may regulate insulin secretion and glucose homeostasis. This study used preproenkephalin knockout mice and their wildtype controls to assess changes in food intake, body weight, and plasma glucose levels when mice were fed a high-fat diet for 16 weeks. At the end of the study, mice were tested using the oral glucose tolerance test and insulin tolerance test. While there were no differences in food intake or body weight between the two genotypes, enkephalin-deficient mice treated with a high-fat diet had reduced insulin sensitivity and impaired oral glucose tolerance test [34]. These results suggest that glucose homeostasis is impaired in the absence of enkephalins, and this may be due to reduced insulin sensitivity.

Finally, the eighth research article investigated the clinical and pharmacotherapeutic profile of patients with type 2 diabetes admitted to a hospital emergency department. People with diabetes are more likely to be admitted to the hospital due to a higher risk of chronic conditions like cardiovascular disease and other metabolic, kidney, and infection-related issues [35]. This study included 420 patients with type 2 diabetes aged ≥65 years using at least one antidiabetic drug and admitted to a hospital emergency department. Those with family support had better glucose control at admission, while patients with obesity had a higher incidence of cardiovascular disease. Those treated with insulin and glucagon-like peptide-1 agonists had a higher incidence of heart failure, while those treated with metformin, with or without dipeptidyl peptidase-4 inhibitors, had a lower incidence of acute kidney injury. Treatment with SGLT2i was associated with hydroelectrolytic disorders [36]. The study results confirmed that adequate social and family support for patients with type 2 diabetes is essential for effective disease management and glycemic control. Moreover, in everyday clinical practice, we must not overlook the potential side effects of therapy.

In conclusion, this Special Issue offers a detailed overview of recent advances in the understanding of hypertension, hypercholesterolemia, and type 2 diabetes; their associated comorbidities; and current therapeutic strategies. This new knowledge will facilitate the diagnosis of these conditions and may be the basis for future therapeutic approaches.
